# Supplementary material for: Cell type composition and circuit organization of clonally related excitatory neurons in the juvenile mouse neocortex
Source: eLife. 2020 Mar 5;9:e52951. doi: 10.7554/eLife.52951 (PMC7162653; doi:10.7554/eLife.52951)
Supplement: Transparent reporting form [file elife-52951-transrepform.pdf]

eLIFE
1st Floor
24 Hills Road
Cambridge CB2 1JP, UK
P 01223 855340
W elifesciences.org
T @elife

eLife's transparent reporting form

We encourage authors to provide detailed information *within their submission* to facilitate the interpretation and replication of experiments. Authors can upload supporting documentation to indicate the use of appropriate reporting guidelines for health-related research (see EQUATOR Network), life science research (see the BioSharing Information Resource), or the ARRIVE guidelines for reporting work involving animal research. Where applicable, authors should refer to any relevant reporting standards documents in this form.

If you have any questions, please consult our Journal Policies and/or contact us: editorial@elifesciences.org.

Sample-size estimation

- You should state whether an appropriate sample size was computed when the study was being designed
- You should state the statistical method of sample size computation and any required assumptions
- If no explicit power analysis was used, you should describe how you decided what sample (replicate) size (number) to use

Please outline where this information can be found within the submission (e.g., sections or figure legends), or explain why this information doesn't apply to your submission:

We could not prospectively estimate an optimal sample size for comparing the distribution of cell types between labeled and unlabeled neurons (Figure 3B) because we did not know *a priori* the fraction of cells that would match to each cell type. To exclude cell types for which we were clearly underpowered to detect differences, we excluded from our analysis cell types with less than three cells total mapped from our data set (Figure 3B). This is reported in the main text (line 314-315) and figure legend (line 344).

Similarly, since we did not know a priori what the baseline probability of unrelated neurons would be for mapping to the same cell type, we could not prospectively estimate the optimal sample size for detecting a difference between related and unrelated neurons. However, after collecting the data and finding that the probability of unrelated neurons matching to the same cell type is approximately 8% (Figure 3C), we can compute the sample size needed to detect a 10% increase in the probability of matching to the same cell type (a conservative estimate of the meaningful effect size) with alpha = 0.05 and 90% statistical power as 233. Given that our sample sizes are 337 and 409 for related and unrelated neurons, respectively, we are appropriately powered to detect a meaningful difference in the probability of related neurons to map to the same transcriptomic cell type. This information is reported in the Materials and Methods section under *Power analysis*.

For our connectivity experiments, we predicted based on a prior study that there would be overall a ~5.7-fold increase in connectivity for clonally related neurons (from 6.3% to 36.3%; Yu et al., 2009). The minimal sample size to detect a difference of this magnitude with 90% statistical power and alpha = 0.05 is 34 in each group. Each group in our study (related and unrelated, for each of nine layer-connection type, a 2x9 matrix) exceeds this sample size, often by two- or three-fold. Thus, we are appropriately powered to capture this previously reported effect size for increased connectivity between clonally related neurons for each layer-specific connection examined. This information is reported in the Materials and Methods section under *Power analysis*.

One possible confound to this estimation could arise if the previously reported connection probabilities only apply to related neurons derived from the same radial glial cell, in particular. In that case, since our clones contain one average two radial glial progenitors, the effect of lineage on connectivity may be diluted, and such an effect could more severely impact lateral connections compared to vertical connections. To address this concern, we performed an extensive power analysis to determine the probability that we would be able to detect a difference in lateral connectivity given this potential dilution effect, which is outlined in detail in the Materials and Methods section under *Power analysis* The results are presented in Figure 5-Supplement 1 and summarized in the main text (lines 475-489).

Replicates

- You should report how often each experiment was performed
- You should include a definition of biological versus technical replication

- The data obtained should be provided and sufficient information should be provided to indicate the number of independent biological and/or technical replicates
- If you encountered any outliers, you should describe how these were handled
- Criteria for exclusion/inclusion of data should be clearly stated
- High-throughput sequence data should be uploaded before submission, with a private link for reviewers provided (these are available from both GEO and ArrayExpress)

Please outline where this information can be found within the submission (e.g., sections or figure legends), or explain why this information doesn't apply to your submission:

This information is reported in detail in the Materials and Methods section, as well as in the figure legends and main text where appropriate. Source Data is provided to support the analyses shown in all of the main figures. The single-cell RNA-seq data is deposited in GEO under accession code GSE140946 and can be accessed using the secure token kbazwegufhohpcb.

Statistical reporting

- Statistical analysis methods should be described and justified
- Raw data should be presented in figures whenever informative to do so (typically when N per group is less than 10)
- For each experiment, you should identify the statistical tests used, exact values of N, definitions of center, methods of multiple test correction, and dispersion and precision measures (e.g., mean, median, SD, SEM, confidence intervals; and, for the major substantive results, a measure of effect size (e.g., Pearson's r, Cohen's d)
- Report exact p-values wherever possible alongside the summary statistics and 95% confidence intervals. These should be reported for all key questions and not only when the p-value is less than 0.05.

Please outline where this information can be found within the submission (e.g., sections or figure legends), or explain why this information doesn't apply to your submission:

The statistical analysis methods are described in detail in the Materials and Methods section. The raw data are presented where appropriate (for clone quantification, in Figure 1 and Figure 1-Supplement 1). Data from different animals (N=3) are shown in different colors in panel 1I,J. The details of each statistical test including test used, N, methods of multiple comparisons correction, dispersion and precision measures are included in the figure legends and the major findings are also reported in the main text.

(For large datasets, or papers with a very large number of statistical tests, you may upload a single table file with tests, Ns, etc., with reference to sections in the manuscript.)

Group allocation

- Indicate how samples were allocated into experimental groups (in the case of clinical studies, please specify allocation to treatment method); if randomization was used, please also state if restricted randomization was applied
- Indicate if masking was used during group allocation, data collection and/or data analysis

Please outline where this information can be found within the submission (e.g., sections or figure legends), or explain why this information doesn't apply to your submission:

For clone quantification (Figure 1), the experimenter was blinded to treatment condition. All library preparation and pre-processing of the transcriptomic data was performed blind to cell identity (labeled or unlabeled). Blinding was not possible at the time of data collection for the connectivity experiments, since the cells are visibly different in color. This information is reported in the Material and Methods section.

Additional data files ("source data")

- We encourage you to upload relevant additional data files, such as numerical data that are represented as a graph in a figure, or as a summary table
- Where provided, these should be in the most useful format, and they can be uploaded as "Source data" files linked to a main figure or table
- Include model definition files including the full list of parameters used
- Include code used for data analysis (e.g., R, MatLab)
- Avoid stating that data files are "available upon request"

Please indicate the figures or tables for which source data files have been provided:

Source Data is provided to support the analyses shown in all of the main figures. The single-cell RNA-seq data is deposited in GEO under accession code GSE140946
